# Prevalence of COVID-19 Vaccine Side Effects among Healthcare Workers in the Czech Republic

**DOI:** 10.3390/jcm10071428

**Published:** 2021-04-01

**Authors:** Abanoub Riad, Andrea Pokorná, Sameh Attia, Jitka Klugarová, Michal Koščík, Miloslav Klugar

**Affiliations:** 1 Department of Public Health, Faculty of Medicine, Masaryk University, Kamenice 5, 625 00 Brno, Czech Republic; klugarova@med.muni.cz (J.K.); koscik@med.muni.cz (M.K.); klugar@med.muni.cz (M.K.); 2 Czech National Centre for Evidence-Based Healthcare and Knowledge Translation (Cochrane Czech Republic, Czech EBHC: JBI Centre of Excellence, Masaryk University GRADE Centre), Institute of Biostatistics and Analyses, Faculty of Medicine, Masaryk University, Kamenice 5, 625 00 Brno, Czech Republic; apokorna@med.muni.cz; 3 Department of Nursing and Midwifery, Faculty of Medicine, Masaryk University, Kamenice 5, 625 00 Brno, Czech Republic; 4 Department of Oral and Maxillofacial Surgery, Justus-Liebig-University, Klinikstrasse 33, 35392 Giessen, Germany; sameh.attia@dentist.med.uni-giessen.de; 5 Czech Clinical Research Infrastructure Network, Department of Pharmacology, Faculty of Medicine, Masaryk University, Kamenice 5, 625 00 Brno, Czech Republic

**Keywords:** adverse effects, BNT162 vaccine, cross-sectional studies, COVID-19, Czech Republic, drug-related side effects and adverse reactions, health personnel, mass vaccination, prevalence

## Abstract

Background: COVID-19 vaccine side effects have a fundamental role in public confidence in the vaccine and its uptake process. Thus far, the evidence on vaccine safety has exclusively been obtained from the manufacturer-sponsored studies; therefore, this study was designed to provide independent evidence on Pfizer–BioNTech COVID-19 vaccine side effects. Methods: A cross-sectional survey-based study was carried out between January and February 2021 to collect data on the side effects following the COVID-19 vaccine among healthcare workers in the Czech Republic. The study used a validated questionnaire with twenty-eight multiple-choice items covering the participants’ demographic data, medical anamneses, COVID-19-related anamneses, general, oral, and skin-related side effects. Results: Injection site pain (89.8%), fatigue (62.2%), headache (45.6%), muscle pain (37.1%), and chills (33.9%) were the most commonly reported side effects. All the general side effects were more prevalent among the ≤43-year-old group, and their duration was mainly one day (45.1%) or three days (35.8%) following the vaccine. Antihistamines were the most common drugs associated with side effects, thus requiring further investigation. The people with two doses were generally associated with a higher frequency of side effects. Conclusions: The distribution of side effects among Czech healthcare workers was highly consistent with the manufacturer’s data, especially in terms of their association with the younger age group and the second dose. The overall prevalence of some local and systemic side effects was higher than the manufacturer’s report. Further independent studies on vaccine safety are strongly required to strengthen public confidence in the vaccine.

## 1. Introduction

Vaccine hesitancy (VH) refers to the “delay in acceptance or refusal of vaccines despite availability of vaccine services”; it is an emerging public health challenge nourished by misinformation related to vaccines effectiveness and safety [1,2,3]. In a recent nation-wide study, aversion to vaccines’ potential side effects was the most frequent cause for VH among population groups in the United Kingdom (U.K.) [4]. This finding was supported in the context of COVID-19 vaccines, because a fear of side effects was the most prominent reason to decrease the readiness of healthcare workers and students in Poland to accept the vaccination [5,6]. Consequently, a systematic review of the strategies of tackling VH revealed that raising public awareness of vaccines’ effectiveness and honesty regarding their side effects is vital for improving vaccine uptake [7]. The launch of the COVID-19 vaccine rollout in December 2020 was a landmark for overcoming this pandemic crisis; therefore, it had been recommended to split the pandemic history to pre-vaccination (B.V.; before vaccine) and post-vaccination (A.V.; after vaccine) eras. COVID-19-related literature should be defined in relation to this parameter either as B.V. or A.V. [8].

In a cross-sectional study of influenza vaccine side effects, three out of thirty-seven participants who were recently influenza-vaccinated (8%) developed oral side effects, thus implying a non-statistically significant relationship between influenza vaccine and the oral cavity [9]. The short-term side effects of vaccines vary in their clinical presentation; however, they are commonly related to prophylactic vaccines’ humoral immune response [10]. The oral cavity has been a locus for the adverse events of an array of vaccines, e.g., diphtheria, tetanus, acellular pertussis, and polio vaccines [9]. The COVID-19-related oral symptoms were attributed to the high expression of angiotensin-converting enzyme 2 (ACE2) receptors in the tongue’s epithelial cells, buccal and gingival mucosa [11,12,13,14,15,16,17,18].

Thus far, all the available data on COVID-19 vaccine side effects has been published by manufacturer-funded studies which are in compliance with the drug authorities’ guidelines and monitored by third-parties [19]. A lack of independent studies on vaccines’ safety may adversely impact the vaccine uptake, which has to be accelerated in the next few months in order to escape this viscous circle of the virus and its variants [7]. Therefore, this study’s primary objective was to estimate the prevalence of Pfizer–BioNTech COVID-19 vaccine side effects among the early vaccinated healthcare workers in the Czech Republic.

The secondary objectives were:

1. To identify the potential risk factors of Pfizer–BioNTech COVID-19 vaccine side effects;
2. To evaluate the correlation of general side effects, oral side effects, and skin-related side effects.

## 2. Materials and Methods

### 2.1. Study Design

This cross-sectional survey-based study was carried out from 27 January 2021 to 27 February 2021, to estimate the prevalence of COVID-19 vaccine side effects among the priority groups of the randomly selected healthcare workers in the Czech Republic. The study utilized a self-administered questionnaire of multiple-choice items which had been designed digitally using KoBoToolbox version 2.021.03 (Harvard Humanitarian Initiative. Cambridge, MA, USA, 2021).

The study protocol was registered in the trials registry of the U.S. National Library of Medicine (NLM) under the title “Oral Side Effects of COVID-19 Vaccine–OSECV” with the identifier NCT04706156; it was reported following the Strengthening the Reporting of Observational Studies in Epidemiology (STROBE) guidelines for cross-sectional studies [20,21].

After ethical clearance, invitation emails for the local coordinators of the member institutions in the Czech Clinical Research Infrastructure Network (CZECRIN; Brno, Czech Republic), the managers of all inpatient healthcare facilities within the network of the Central Adverse Events Reporting System of the Institute of Health Information and Statistics of the Czech Republic (IHIS-CR; Prague, Czech Republic), and all registered dentists through the Czech Dental Chamber (ČSK; Prague, Czech Republic) to contribute to this study by accessing the uniform resource locator (URL) of the digital questionnaire [22]. The awareness of the study was also raised by promotion on the websites and social media profiles of CZECRIN and the Faculty of Medicine [23]. The collected data are controlled by Masaryk University, and data acquisition and processing are in compliance with the General Data Protection Regulation (GDPR) [24].

### 2.2. Participants

Inclusion criteria for this study were Czech healthcare workers who were vaccinated with the Pfizer–BioNTech COVID-19 vaccine during the early vaccination phase of the governmental strategy (Phase 1A) [25]. The eligible participants should have received the latest dose of the vaccine, either the first or the second dose, no more than thirty days before filling in the questionnaire. Non-healthcare workers who were vaccinated during Phase 1A and the healthcare workers who were vaccinated in February 2021 by Moderna COVID-19 vaccine and Oxford/AstraZeneca COVID-19 vaccine were excluded from this report. Participation in this study was not compensated financially or by any other incentives.

### 2.3. Instrument

The self-administered questionnaire of this study was composed of twenty mandatory multiple-choice items and eight conditional multiple-choice items, and it was adapted from previous studies on the oral side effects of various vaccines by the authors [9,26]. A panel of four experts in oral medicine, maxillofacial surgery, and infectious diseases were formed to review the questionnaire draft and to assess its content validity. We used an iterative discussion to finalize the questionnaire. Later, the reliability of the questionnaire was evaluated by a group of eighteen recently vaccinated healthcare workers, who filled in the questionnaire twice with a minimum interval of two weeks. The result of the test re-test of the provisional instrument yielded substantial reliability, with a mean Cohen’s kappa coefficient of 0.89 ± 0.13 (0.54–1) (Table 1).

The questionnaire was divided into four main categories: (i) demographic data including gender, age, region, profession, and length of work experience; (ii) medical anamnesis including medical comorbidities and medications; (iii) COVID-19 related anamnesis including vaccination date and the number of doses, previous infection, and exposure to infected cases; and (iv) vaccine side effects including general side effects, oral side effects, and skin-related side effects (Supplementary Materials Table S1).

### 2.4. Ethical Considerations

The study was reviewed and approved by the Ethics Committee of the Faculty of Medicine at Masaryk University on 20 January 2021 (Ref. 2/2021). Digital informed consent had been obtained from each participant prior to participation. The participants were allowed to withdraw from the study at any moment without justifying, and no data were saved before the participant submitted their answers completely.

### 2.5. Statistical Analysis

All statistical tests were executed using the Statistical Package for the Social Sciences (SPSS) version 27.0 (SPSS Inc. Chicago, IL, USA, 2020). Primarily, descriptive statistics were carried out for the demographic variables (gender, age, profession, length of work experience, and region), and medical anamnesis (non-communicable diseases, and medical treatments), COVID-19-related anamnesis (number of doses, interval between doses, previous infection, patency period, and previous exposure to COVID-19 cases), and vaccine side effects (general side effects, oral side effects, and skin-related side effects) were represented by frequencies, percentages, means and standard deviations. Consequently, inferential statistics were performed to assess the association between side effects and medical anamnesis, and the association of various side effects and each other using the chi-squared test (*χ*^2^), Student’s *t*-test, one-way analysis of variance (ANOVA), and Pearson’s correlation test (*r*), with a confidence level of 95% and significance value *p* ≤ 0.05. Strengths of correlation are verbally described by the value of (*r*) 0.00–0.19 “very weak”; 0.20–0.39 “weak”; 0.40–0.59 “moderate”; 0.60–0.79 “strong”; 0.80–1.0 “very strong”.

## 3. Results

### 3.1. Demographic Characteristics

A total of 922 participants filled in the questionnaire properly by 27 February 2021. Nineteen participants were administrative staff at healthcare facilities; therefore, they were vaccinated, but they did not meet the study’s inclusion criteria. Similarly, twenty-eight participants received either the Moderna COVID-19 vaccine or Oxford/AstraZeneca COVID-19 vaccine; therefore, they were excluded from this report. Three participants did not submit their age properly; therefore, they were omitted from the inferential statistics based on age groups.

A total of 877 participants were included in the final analyses; 776 (88.5%) were females, 100 (11.4%) were males, and 1 (0.1%) preferred not to state their gender. Their mean age was 42.56 ± 10.5 years old, and it ranged between 19 and 78 years old with a median of 43 years old. Given the fact that the median age of this study’s participants corresponds with the mean age of healthcare workers in the Czech Republic, which is between 40 and 45 years old, the sample’s median age (43 years old) had been used as a cut-off to present the anamnestic characteristics and the COVID-19 vaccine side effects of the participants [28,29] (Table 2).

**Figure 1:**
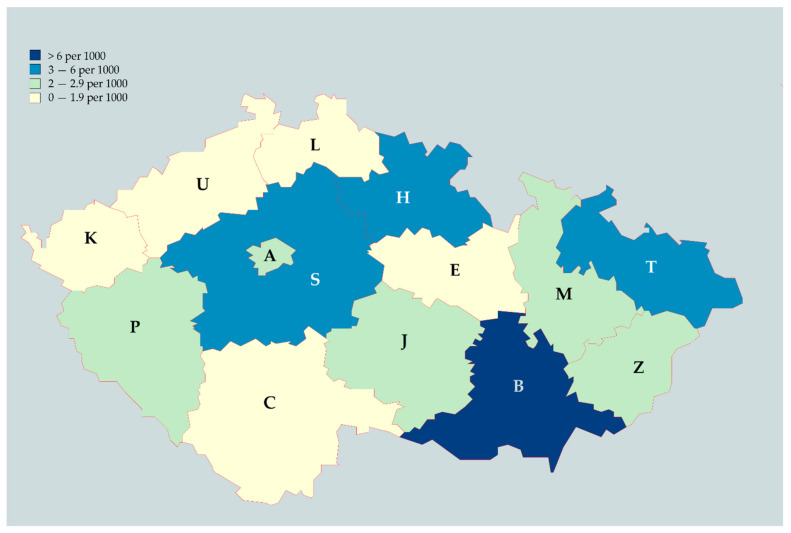
Regional density of the participating Czech healthcare workers; (A) Prague; (S) Central Bohemian; (C) South Bohemian; (J) Vysočina; (P) Plzen; (K) Karlovy Vary; (U) Usti nad Labem; (L) Liberec; (H) Hradec Kralove; (E) Pardubice; (M) Olomouc; (T) Moravian-Silesian; (B) South-Moravian; (Z) Zlin; January–February 2021.

On comparing the number of participants to the total number of healthcare workers per region, reported by IHIS-CR, the mean density was 2.95 ± 2.22 responses per 1000 healthcare workers [30]. The highest density was in the South-Moravian region with 9.86 response per 1000 healthcare workers; the lowest was in the South Bohemian region with 1.51 response per 1000 healthcare workers (Figure 1).

### 3.2. Medical Anamneses

A total of 271 (31%) participants reported having at least one non-communicable disease (NCD) with a statistically significant difference between the ≤43-year-old group and the >43-year-old group: 105 (23.9%) vs. 166 (38.2%), respectively. Out of all the chronically ill participants, 100 (36.9%) reported chronic hypertension, 69 (25.6%) thyroid disease, 59 (21.8%) asthma, 23 (8.5%) diabetes mellitus type-2, 16 (5.9%) cardiac disease, 16 (5.9%) allergy, 13 (4.8%) rheumatoid arthritis, 12 (4.4%) bowel disease, 11 (4.1%) blood disease, 11 (4.1%) neurologic disease, 8 (3%) psychological distress, 6 (2.2%) renal disease, 5 (1.8%) chronic obstructive pulmonary disease (COPD), 4 (1.5%) cancer, 3 (1.1%) diabetes mellitus type-1, 2 (0.7%) hepatologic disease, and 1 (0.4%) ophthalmologic disease. Across the age groups, the total number of NCDs was significantly higher in the >43-year-old group (1.39 ± 0.66) than the ≤43-year-old group (1.22 ± 0.46) with a significance value of 0.020 (Table 3).

The chi-squared test revealed a statistically significant difference in the distribution of some NCDs between both age groups, e.g., chronic hypertension, psychological distress, blood disease, and diabetes mellitus type-2 with significance values less than 0.0001, 0.004, 0.018, and 0.028, respectively.

A total of 384 (44%) participants reported receiving at least one medical treatment at the time of filling in the questionnaire, with a statistically significant difference between the ≤43-year-old group and the >43-year-old group: 144 (32.8%) vs. 240 (55.2%), respectively. Out of all the regularly taken drugs, antihypertensive drugs were taken by 98 (25.5%), followed by thyroid hormones replacement by 90 (23.4%), antihistamine by 75 (19.6%), antidepressant by 45 (11.7%), contraceptives by 21 (5.5%), common analgesics 19 (4.9%), nonsteroidal anti-inflammatory (NSAID) by 15 (3.9%), antidiabetics by 14 (3.6%), anti-reflux by 13 (3.4%), cholesterol-lowering by 12 (3.1%), immunosuppressive by 8 (2.1%), anti-asthma by 7 (1.8%), venous insufficiency by 6 (1.6%), anticoagulants by 6 (1.6%), antiepileptics by 6 (1.6%), corticosteroids by 6 (1.6%), opioid analgesics by 3 (0.8%), antibiotics by 2 (0.5%), and other drugs by 18 (4.7%), including bronchodilators, antifungals, antidiuretic, estrogen hormone, chemotherapy, vitamin D, and interferon. Across the age groups, the total number of taken drugs was insignificantly lower in the >43-year-old group (1.20 ± 0.51) than the ≤43-year-old group (1.24 ± 0.55) (Table 4).

The chi-squared test revealed a statistically significant difference in the distribution of some taken drugs between both age groups, e.g., antihypertensive drugs, contraceptives, and antihistamine drugs, with significance values of 0.001, 0.001, and 0.018, respectively.

### 3.3. COVID-19-Related Anamnesis

By the time of filling in the questionnaire, the vast majority of the participants had received both doses of the Pfizer–BioNTech COVID-19 vaccine (93.6%), while 56 (6.4%) had received the first dose only. The interval between the first dose and the second dose ranged between 7 and 55 days, with a median of 21 days. The difference was statistically insignificant across the age groups, with a slight longer interval among the ≤43-year-old group (22.69 ± 5.14 days) compared to the >43-year-old group (22.48 ± 4.7 days).

Although 169 (19.3%) participants had been previously infected by COVID-19, the patency period between the recovery date and the first vaccine dose ranged between 7 and 270 days with a median of 65 days. Regarding the exposure to COVID-19 cases, a total of 617 (70.6%) participants reported that they had been in contact with COVID-19 cases previously: 317 (72.2%) of the ≤43-year-old group and 300 (69%) of the >43-year-old group, without statistical significance (*p* = 0.293) (Table 5).

### 3.4. COVID-19 Vaccine Reported Side Effects

#### 3.4.1. Prevalence of General Side Effects

A total of 814 (93.1%) participants reported having at least one side effect following the COVID-19 vaccine. The prevalence of side effects was slightly higher in the ≤43-year-old group (94.8%) than the >43-year-old group (91.5%). The most common side effect was injection site pain (89.8%), followed by fatigue (62.2%), headache (45.6%), muscle pain (37.1%), and chills (33.9%). All the reported side effects were more prevalent in the ≤43-year-old group than the >43-year-old group, with a statistically significant difference in the case of injection site pain (93.3% vs. 86.2%), headache (50.7% vs. 40.2%), fatigue (65.9% vs. 58.3%), muscle pain (40.9% vs. 33.2%), and feeling unwell (26% vs. 19.8%).

Regarding the general side effects’ duration, 45.1% of them lasted for 1 day, while 35.8% lasted for 3 days, 9.4% lasted for 5 days, 5.3% lasted for one week, 3% lasted for over a week, and 1.4% for over a month. The severe side effects that required medical intervention was reported by only 1.3% of the whole study group (Table 6).

#### 3.4.2. Prevalence of Reported Oral Side Effects

A total of 114 (13%) participants reported to have at least one oral side effect following the Pfizer–BioNTech COVID-19 vaccine. The prevalence of oral side effects was insignificantly higher in the ≤43-year-old group (13.4%) than the >43-year-old group (12.6%). The most common oral side effect was blisters (36%), followed by halitosis (25.4%), ulcers (14%), bleeding gingiva (11.4%), and white/red plaque (10.5%).

However, there were no statistically significant differences between the age groups: white/red plaque (10.9% vs. 10.2%), burning gingiva (9.1% vs. 8.5%), angular cheilitis (5.5% vs. 3.4%), tongue tingling (5.5% vs. 3.4%), taste disturbance (5.5% vs. 1.7%), vesicles (3.6% vs. 3.4%), and xerostomia (3.6% vs. 1.7%) were more prevalent in the >43-year-old group than the ≤43-year-old group. On the other hand, blisters (37.3% vs. 34.5%), halitosis (28.8% vs. 21.8%), ulcers (16.9% vs. 10.9%), bleeding gingiva (13.6% vs. 9.1%), and swollen lips (5.1% vs. 1.8%) were more prevalent in the ≤43-year-old group compared to the >43-year-old group.

Regarding oral side effects’ onset, 28.6% of them emerged within the first week post-vaccination, while 26.8% emerged within 1–3 days post-vaccination, 18.8% within the third week post-vaccination, 16.1% within the second week post-vaccination, and 9.8% within the fourth week post-vaccination.

The most common location of ulcers, blisters, and vesicles was the lips (74.1%), followed by labial and buccal mucosa (14.8%), tongue (13%), palate (9.3%), and gingiva (9.3%). The difference between the age groups was statistically insignificant; however, lips were affected in 80% of the >43-year-old group versus 69% of the ≤43-year-old group. All (100%) of the >43-year-old group participants had one affected location, whereas 72.4% of the ≤43-year-old group participants had one affected location, 20.7% had two affected locations, 3.4% had three affected locations, and 3.4% had four affected locations. In the case of white/red plaque, the most common location was the tongue dorsum (75%), followed by soft palate (16.7%) and labial and buccal mucosa (8.3%) (Table 7).

#### 3.4.3. Prevalence of Skin-Related Side Effects

A total of 45 (5.2%) participants reported having at least one skin-related side effect following the COVID-19 vaccine. The prevalence of skin-related side effects was insignificantly higher in the ≤43-year-old group (6.2%) than the >43-year-old group (4.1%). The most common skin-related side effect was rash (62.2%), followed by urticaria (22.2%), and other non-specific conditions (20%). Upper limb was the most common location (60%); chest and trunk were the second most common (33.3%), and it was more common among the older age group than the younger age group (Table 8).

#### 3.4.4. COVID-19 Vaccine Side Effects and Medical Anamneses

The correlation test between the composite variables of side effects and medical anamnesis revealed a significant direct association between the total number of general side effects and the total number of medical treatments (*r =* 0.108; *p* = 0.041). Age was significantly inversely correlated with the total number of general side effects (*r =* −0.180; *p <* 0.001).

The general side effects’ duration was significantly and directly correlated with age (*r =* 0.097; *p* = 0.006), the total number of medical treatments (*r =* 0.122; *p* = 0.021), and the total number of general side effects (*r =* 0.256; *p <* 0.001). The total number of NCDs was directly correlated with age (*r =* 0.182; *p* = 0.003), the total number of medical treatments (*r =* 0.232; *p <* 0.001), and the total number of general side effects (*r =* 0.072; *p* = 0.258).

Similarly, the oral side effects were inversely, but not significantly, correlated with age (*r =* −0.164; *p* = 0.086). The total number of oral side effects was positively correlated with the total number of NCDs (*r =* 0.045; *p* = 0.790), the total number of medical treatments (*r =* 0.175; *p* = 0.188), the total number of general side effects (*r =* 0.202; *p* = 0.038), and the general side effects’ duration (*r =* 0.279; *p* = 0.004).

The oral side effects’ onset was inversely correlated with the (*r =* −0.202; *p* = 0.033), and directly correlated with the total number of NCDs (*r =* 0.018; *p* = 0.914), the total number of medical treatments (*r =* 0.168; *p* = 0.208), the general side effects’ duration (*r =* 0.025; *p* = 0.794), and the total number of oral side effects (*r =* 0.143; *p* = 0.138) (Table 9).

#### 3.4.5. Risk Factors of COVID-19 Vaccine Side Effects

Injection site pain was significantly more prevalent among the younger age group (*p* = 0.001), the healthcare workers with shorter work experience (*p* = 0.009), the participants with diabetes mellitus type-2 (*p* = 0.019), and the participants receiving antidiabetic drugs (*p* = 0.038) and venous insufficiency drugs (*p* = 0.028).

Injection site swelling was significantly more prevalent among females (*p* = 0.021), the participants receiving corticosteroids (*p* = 0.028), and the previously infected participants (*p* = 0.030).

Injection site redness was significantly more prevalent among the participants with allergies (*p* = 0.018), and the participants receiving antihistamine drugs (*p* = 0.013), and corticosteroids (*p* = 0.001). It was also significantly associated with the total number of NCDs (*p* = 0.010), and the total number of medical treatments (*p* = 0.031).

Fatigue was significantly more prevalent among the young age group (*p* = 0.024), the healthcare workers with shorter work experience (*p* = 0.026), and the participants not receiving cholesterol-lowering drugs (*p* = 0.010).

Headache was significantly more prevalent among females (*p* = 0.006), the young age group (*p* = 0.003), the healthcare workers with shorter work experience (*p* = 0.007), and the participants receiving antihistamine drugs (*p* = 0.007).

Nausea was significantly more prevalent among females (*p* = 0.015), the healthcare workers with shorter work experience (*p* = 0.029), and the participants receiving antihistamine drugs (*p* = 0.027), and antidepressants (*p* = 0.013).

Feeling unwell was significantly more prevalent among the younger age group (*p* = 0.032), the healthcare workers with shorter work experience (*p* = 0.003), and the participants with hepatologic disease (*p* = 0.006) and renal disease (*p* = 0.008).

Muscle pain was significantly more prevalent among the younger age group (*p* = 0.025), the participants receiving antidepressants (*p* = 0.053) and antiepileptics (*p* = 0.018), and the previously exposed participants to COVID-19 (*p* = 0.007).

Joint pain was significantly more prevalent among the participants with NCDs (*p* = 0.041) and hepatologic disease (*p* = 0.041), the participants receiving antibiotics (*p* = 0.034) and antidepressants (*p* = 0.021), and the previously exposed participants (*p* = 0.027).

Fever was significantly more prevalent among the healthcare workers with shorter work experience (*p* = 0.014), the participants with NCDs (*p* = 0.033) and asthma (*p* = 0.008), the participants receiving antihistamine drugs (*p* = 0.011) and NSAIDs (*p* = 0.035), and the previously infected participants (*p* = 0.023).

Chills were significantly more prevalent among the healthcare workers with shorter work experience (*p* = 0.005), and the participants receiving antihistamine drugs (*p* = 0.014) and NSAIDs (*p* = 0.029).

Lymphadenopathy was significantly more prevalent among the participants receiving antihistamine drugs (*p* = 0.019).

#### 3.4.6. Number of Doses and Side Effects’ Prevalence

The prevalence of injection site pain, swelling, redness, fatigue, headache, nausea, muscle pain, lymphadenopathy was higher among the participants who received two doses compared to the participants with one dose. Injection site redness was the only general side effect that was significantly more prevalent in the two-doses group (23.9%) than the one-dose group (8%), with a *p*-value of 0.10.

The oral side effects were insignificantly more prevalent in the one dose group, e.g., ulcers, white/red plaque, and bleeding gingiva. In contrast, vesicles, blisters, burning gingiva, swollen lips, angular cheilitis, xerostomia, taste disturbance, and tongue tingling were more common in the two-dose group. The mean total number of oral side effects was higher in the one-dose group (Table 10).

#### 3.4.7. Oral and General Side Effects of COVID-19 Vaccine

The emergence of oral side effects was significantly associated with some general side effects, e.g., headache (*χ*^2^ = 13.18; *p* < 0.001), nausea (*χ*^2^ = 10.36; *p* = 0.001), muscle pain (*χ*^2^ = 4.56; *p* = 0.033), fever (*χ*^2^ = 4.86; *p* = 0.027), and lymphadenopathy (*χ*^2^ = 9.78; *p* = 0.002). In addition to the association between the total number of general side effects, their duration, and the emergence of oral side effects, blisters were significantly lower among the participants receiving thyroid hormone replacements (*χ*^2^ = 4.05; *p* = 0.044). In contrast, angular cheilitis was significantly more prevalent among the participants receiving thyroid hormone replacements (*χ*^2^ = 7.2; *p* = 0.007).

## 4. Discussion

The first evidence to evaluate the efficacy of the Pfizer–BioNTech COVID-19 vaccine was obtained from a randomized controlled trial (RCT) which recruited 43,000 volunteers with a median age of 52 years old. The early results of this RCT showed that the vaccine’s efficacy was around 95%, with several adverse reactions that occurred in the few days following the vaccine shot [19]. The vaccine’s side effects could be categorized as either local or systemic reactions, and their severity varied from mild to moderate [31].

The present study reported a statistically significant difference in the prevalence of injection site pain (*p* = 0.001), fatigue (*p* = 0.026), headache (*p* = 0.003), muscle pain (*p* = 0.023), and feeling unwell (*p* = 0.038) between the ≤43-year-old group and the >43-year-old group, where the younger adults were more frequently affected. These findings are consistent with those reported by the Food and Drug Administration (FDA): injection site pain was more prevalent in the ≤55-year-old group than the >55-year-old group (80.56% vs. 68.75%); fatigue was more prevalent in the younger group (53.13% vs. 42%), headache was also more prevalent in the younger group (46.57% vs. 31.8%); and muscle pain was more prevalent in the younger group (28.94% vs. 21.03%) [19].

However, differences between the age groups in terms of fever, chills and joint pain were not statistically significant in our sample: the ≤43-year-old group was more affected by fever (9.47% vs. 5.98%), chills (36.8% vs. 30.9%) and joint pain (28.6% vs. 26.9%) than the >43-year-old group. These trends were similar to the FDA’s report, where fever was more prevalent among young adults than old adults (24.3% vs. 19.1%), and the same pattern was recorded for chills (24.11% vs. 14.15%) and joint pain (16.18% vs. 13.52%). In contrast to the manufacturer’s data, injection site swelling (26% vs. 25.2%) and injection site redness (25.5% vs. 20.4%) were more frequent in the younger age group of our sample. According to the FDA’s report, injection site swelling was slightly less frequent among the younger adults (6.02% vs. 7.51%). Injection site redness was also slightly less frequent among the younger adults (5.37% vs. 5.92%).

The overall frequency of systemic reactions including fever, fatigue, headache, chills, vomiting, diarrhea, muscle pain and joint pain was significantly higher among the younger adults than the older adults, according to the FDA’s report (82.8% vs. 70.6%). The same pattern was reported for local reactions, including injection site pain, swelling, and redness, where 88.7% of younger adults were affected compared to 79.7% of the older. This pattern was identified in our sample; the mean number of side effects (4.50 ± 2.596 vs. 3.87 ± 2.599) and the overall frequency of affected participants (94.8% vs. 91.5%) were significantly higher in the younger than the older.

The overall frequencies of injection site pain (89.8% vs. 75.35%), injection site swelling (25.6% vs. 6.44%), and injection site swelling (23% vs. 5.5%) were significantly higher among the Czech healthcare workers than the volunteers of the Pfizer–BioNTech trial [19]. In contrast, the overall frequency of headache was quite consistent between the Czech sample and the FDA’s report (45.6% vs. 40.06%, respectively).

On comparing the first dose and the second dose of the vaccine, the FDA’s report revealed that the frequency of local side effects was slightly higher after the second dose compared to the first dose. The same trend was more significant in the case of systemic side effects [19]. The Czech data confirmed this trend in all the reported side effects except for injection site redness, which was more frequent among the people with one dose only.

Injection site pain as a subjectively reported symptom has a number of confounders that are worth being considered for future research on vaccines’ side effects, including injection technique, vaccine temperature, and injection velocity. These confounders are difficult to be standardized and will significantly impact one’s experience [32]. Moreover, injection in a relaxed muscle leads to less pain compared to a tensed one; therefore, it is recommended to lower the patient’s arm which will be injected. Vaccines in situ are preserved in very low temperature, including the BNT162b2 vaccine which requires −70 °C, and if injected without optimal warming up, this may increase the probability of post-vaccination pain of the injection site [33]. Additionally, muscle mass might play a role in pain perception following the injection. The healthcare workers involved in the vaccination process are highly recommended to receive appropriate training on optimal injection techniques to reduce inequalities in patients’ experience of pain after vaccination [34].

The allergic population that used antihistamine drugs were the most susceptible group for experiencing side effects, because they were significantly affected by injection site redness (*χ*^2^ = 6.27; *p* = 0.012), headache (*χ*^2^ = 7.5; *p* = 0.006), nausea (*χ*^2^ = 4.97; *p* = 0.026), fever (*χ*^2^ = 6.62; *p* = 0.01), chills (*χ*^2^ = 6.1; *p* = 0.014), and lymphadenopathy (*χ*^2^ = 5.54; *p* = 0.019). The Centers for Disease Control and Prevention (CDC) had stated, within its interim guidelines for COVID-19 vaccine rollout, that people with a history of any immediate allergic reaction to other vaccines or injectable therapies should be vaccinated with high precaution. People with a history of severe allergic reactions such as anaphylaxis after a previous dose or to a component of the vaccine such as polyethylene glycol (PEG) are prohibited from receiving the vaccine at this stage [31]. Although people with allergies to oral medications, food, pets, insects, venom, latex, and other environmental insults and family histories are recommended to proceed with receiving the vaccine normally, it is worth noting that antihistamine consumption increases considerably in spring in Europe; therefore, special attention should be given to the prescription of these drugs during this season in the context of vaccination. This result will be further explored in our upcoming study phase.

Lymphadenopathy of the arm and neck was among the unsolicited side effects in the FDA’s report with 64 cases; however, it should have been a predictable side effect due to it being common with other vaccines such as the human papillomavirus vaccine and influenza vaccine [35,36,37] Therefore, in this study, lymphadenopathy was among the general side effects of the COVID-19 vaccine and its overall prevalence was 16.2%, with a higher frequency among females compared to males (16.8% vs. 10.6%), young adults compared to old adults (17.3% vs. 15.1%), and people with allergies (21.4% vs. 14.5%), asthma (17.6% vs. 14.1%), bowel disease (25% vs. 14.3%), cardiac disease (25% vs. 14.2%), COPD (20% vs. 14.8%), DM type-2 (19% vs. 14.5%), and neurologic disease (30% vs. 14.2%). The majority of participants with lymphadenopathy reported that its duration was either one day (18.9%), three days (43.9%), or five days (18.2%). This finding is slightly in agreement with the FDA’s report, where lymphadenopathy emerged 2–4 days post-vaccination and lasted for approximately 10 days.

The median interval between the first dose and the second dose was 21 days, which is in compliance with the recommended interval of the Czech ministry of health (MOH) [38]. The median patency period between the recovery date and the first vaccine dose was 65 days, which fulfills the recommendation of the Czech MOH for a patency period of seven days between the positive test and the vaccination [39].

The reported NCDs in our sample were generally less frequent than what is reported for the general Czech population. This difference was predictable for this special subset of the population, because medical fitness is a prerequisite for pursuing healthcare professions. Unfortunately, the data on diseases prevalence in the Czech Republic are not stratified by profession or employment sector; therefore, there is no reference prevalence for Czech healthcare workers. Diabetes Mellitus type-2 had prevalence in the Czech Republic around 7.4% (2017); however, in our sample, its prevalence was considerably lower 2.63% [40,41]. Cardiac disease and chronic hypertension had prevalence around 4.3% and 23.7%, respectively (2019), which were two-fold higher than the prevalence values of our sample, which were 1.83% and 11.44%, respectively [42,43]. In contrast, asthma had a prevalence in the Czech Republic 4.5% (2018), while in our sample it was 6.75% [44]. Thyroid disease had a prevalence in the Czech Republic in 7.5% (2015) which was similar to our sample (7.89%) [45].

### 4.1. Strengths and Limitations

The findings of this study should be interpreted cautiously regarding the association of side effects with the second dose of the vaccine, because we did not ask whether the side effect occurred after the first dose or the second dose. The external validity of this study is limited because the sample was not equally distributed across gender or profession. Another methodological limitation is due to the survey-based technique that may lead to self-selection bias, when perhaps only the highly motivated participants filled in the questionnaire. The self-reporting nature of the collected data compromises its objectivity when it comes to clinical evaluation and standardization. This methodological confounding had been controlled to some degree because all the study’s participants were healthcare workers who have a high level of health literacy and medical expertise, so the outcomes were supposed to be accurately reported. To the best of our knowledge, this was the first independent study dealing with the BNT162b2 vaccine side effects, and the first designed study evaluating the side effects among a European population.

### 4.2. Study Implications

1. Further independent (non-sponsored) epidemiological studies for COVID-19 vaccine side effects should be carried out by academic institutions in the upcoming months to increase public confidence in the vaccines’ safety and accelerate its uptake process.
2. The upcoming studies will benefit from comparing data of different vaccines from other manufacturers.
3. The upcoming studies of vaccine side effects should distinguish between the side effects that emerged after the first dose, the second dose, and both doses.
4. Healthcare workers and healthcare students are among the ideal population groups to participate in this type of studies due to their high level of health literacy and scientific motivation.
5. The potential association between antihistamine drugs and the vaccine side effects’ frequency should be further explored.

## 5. Conclusions

The most common side effects of the Pfizer–BioNTech COVID-19 vaccine among Czech healthcare workers were injection site pain, fatigue, headache, muscle pain, chills, and joint pain. They were highly consistent with the data reported by the manufacturer in terms of their association with the younger age group and the second dose. The overall prevalence of some local and systemic side effects was higher than the manufacturer’s report; this could be attributed to the special type of population enrolled in this study. Antihistamines were the most common drugs associated with side effect emergence, which might require special attention in the following months. The oral side effects were significantly associated with headache, nausea, muscle pain, fever, and lymphadenopathy. Further independent studies on vaccine safety are strongly required to strengthen the public confidence in the vaccine, and to provide a better understanding of the potential risk factors of vaccine side effects.

## Figures and Tables

**Table 1:** The results of test re-test reliability of the instrument of the “Oral Side Effects of COVID-19 Vaccine” study (OSECV) ^1^.

Participant	*κ* Coefficient	Participant	*κ* Coefficient
No. 1	0.821	No. 10	0.540
No. 2	0.842	No. 11	1.000
No. 3	0.777	No. 12	1.000
No. 4	0.940	No. 13	1.000
No. 5	1.000	No. 14	1.000
No. 6	1.000	No. 15	0.937
No. 7	0.934	No. 16	0.872
No. 8	0.758	No. 17	0.868
No. 9	1.000	No. 18	0.762

^1^ Cohen’s Kappa statistic (*κ*): 0.01–0.20 as none to slight; 0.21–0.40 as fair; 0.41–0.60 as moderate; 0.61–0.80 as substantial; and 0.81–1.00 as perfect agreement [27].

**Table 2:** Demographic characteristics of the Czech healthcare workers who received the Pfizer–BioNTech COVID-19 vaccine, January–February 2021.

Variable	Outcome	Frequency	Percentage
**Gender**	Female	776	88.5%
	Male	100	11.4%
	Prefer not to say	1	0.1%
**Age**	≤43 years old	439	50.2%
	>43 years old	435	49.8%
**Profession**	Registered Nurse	540	61.6%
	Physician	77	8.8%
	Practice Nurse	75	8.6%
	Lab Worker	46	5.2%
	Paramedic	26	3.0%
	Dentist	24	2.7%
	Midwife	23	2.6%
	Pharmacist	21	2.4%
	Physiotherapist	19	2.2%
	Radiological Assistant	12	1.4%
	Psychologist	8	0.9%
	Dietitian	5	0.6%
	Dental Hygienist	1	0.1%
**Length of Work Experience**	1–5 years	134	15.3%
	6–10 years	88	10.0%
	11–20 years	188	21.4%
	>20 years	467	53.2%
**Region**	South-Moravian	301	34.3%
	Prague	105	12.0%
	Moravian-Silesian	92	10.5%
	Hradec Kralove	78	8.9%
	Central Bohemian	70	8.0%
	Olomouc	51	5.8%
	Plzen	29	3.3%
	Usti nad Labem	29	3.3%
	Zlin	25	2.9%
	Vysočina	23	2.6%
	South Bohemian	22	2.5%
	Pardubice	21	2.4%
	Karlovy Vary	17	1.9%
	Liberec	14	1.6%

**Table 3:** Non-communicable diseases of vaccinated healthcare workers in the Czech Republic, January–February 2021.

Disease	≤43 Years Old	>43 Years Old	Total	Significance ^1^
Allergy	9 (8.6%)	7 (4.2%)	16 (5.9%)	0.138
Asthma	26 (24.8%)	33 (19.9%)	59 (21.8%)	0.343
Blood Disease	8 (7.6%)	3 (1.8%)	11 (4.1%)	**0.018**
Bowel Disease	7 (6.7%)	5 (3.0%)	12 (4.4%)	0.154
Cancer	0 (0.0%)	4 (2.4%)	4 (1.5%)	0.109
Cardiac Disease	4 (3.8%)	12 (7.2%)	16 (5.9%)	0.245
Chronic Hypertension	21 (20.0%)	79 (47.6%)	100 (36.9%)	**<0.0001**
COPD	0 (0.0%)	5 (3.0%)	5 (1.8%)	0.073
Diabetes Mellitus–I	2 (1.9%)	1 (0.6%)	3 (1.1%)	0.318
**Diabetes Mellitus–II**	4 (3.8%)	19 (11.4%)	23 (8.5%)	**0.028**
Hepatologic Disease	2 (1.9%)	0 (0.0%)	2 (0.7%)	0.074
**Psychological Distress**	7 (6.7%)	1 (0.6%)	8 (3.0%)	**0.004**
Neurologic Disease	2 (1.9%)	9 (5.4%)	11 (4.1%)	0.153
Ophthalmologic Disease	0 (0.0%)	1 (0.6%)	1 (0.4%)	0.426
Renal Disease	2 (1.9%)	4 (1.8%)	6 (2.2%)	0.783
Rheumatoid Arthritis	3 (2.9%)	10 (6%)	13 (4.8%)	0.235
Thyroid Disease	31 (29.5%)	38 (22.9%)	69 (25.5%)	0.222
**Number of NCDs (1–17)**	1.22 ± 0.46	1.39 ± 0.66	1.32 ± 0.59	**0.026**
**Total**	105 (23.9%)	166 (38.2%)	271 (31%)	**0.020**

^1^ Chi-square test and Student’s *t*-test were used with a significance level of <0.05. Bold format highlight the significantly different diseases across age groups.

**Table 4:** Regularly taken drugs by the vaccinated healthcare workers in the Czech Republic, January–February 2021.

Drug	≤43 Years Old	>43 Years Old	Total	Significance ^1^
Anti-asthma	4 (2.8%)	3 (1.3%)	7 (1.8%)	0.279
Antibiotics	1 (0.7%)	1 (0.4%)	2 (0.5%)	0.714
Anticoagulant	1 (0.7%)	5 (2.1%)	6 (1.6%)	0.288
Antidepressant	19 (13.2%)	26 (10.8%)	45 (11.7%)	0.486
Antidiabetic	3 (2.1%)	11 (4.6%)	14 (3.6%)	0.206
Antiepileptic	4 (2.8%)	2 (0.8%)	6 (1.6%)	0.138
**Antihistamine**	37 (25.7%)	38 (15.8%)	75 (19.5%)	**0.018**
**Antihypertensive**	23 (16.0%)	75 (31.3%)	98 (25.5%)	**0.001**
Anti-Reflux	6 (4.2%)	7 (2.9%)	13 (3.4%)	0.512
Immunosuppressive	2 (1.4%)	6 (2.5%)	8 (2.1%)	0.460
Cholesterol-lowering	3 (2.1%)	9 (3.8%)	12 (3.1%)	0.363
Common Analgesic	7 (4.9%)	12 (5.0%)	19 (4.9%)	0.952
**Contraceptive**	15 (10.4%)	6 (2.5%)	21 (5.5%)	**0.001**
Corticosteroid	2 (1.4%)	4 (1.7%)	6 (1.6%)	0.832
Nonsteroidal anti-inflammatory (NSAID)	6 (4.2%)	9 (3.8%)	15 (3.9%)	0.838
Opioid Analgesic	2 (1.4%)	1 (0.4%)	3 (0.8%)	0.295
Thyroid Hormones	35 (24.3%)	55 (22.9%)	90 (23.4%)	0.756
Venous Insufficiency	4 (2.8%)	2 (0.8%)	6 (1.6%)	0.137
Other	5 (3.5%)	13 (5.4%)	18 (4.7%)	0.383
Number of Drugs (1–19)	1.24 ± 0.55	1.20 ± 0.51	1.21 ± 0.52	0.392
**Total**	144 (32.8%)	240 (55.2%)	384 (43.9%)	<0.0001

^1^ Chi-squared test and ANOVA were used with a significance level of <0.05.

**Table 5:** COVID-19-related anamnesis of vaccinated healthcare workers in the Czech Republic, January–February 2021.

Variable	Outcome	≤43 Years Old	>43 Years Old	Total	Significance ^1^
**Number of doses**	One dose	26 (5.9%)	30 (6.9%)	56 (6.4%)	0.557
	Two doses	413 (94.1%)	404 (93.1%)	818 (93.6%)	
**Interval**	(days)	22.69 ± 5.14	22.48 ± 4.7	22.58 ± 4.92	0.551
**COVID-19 infection**	Yes	90 (20.5%)	79 (18.2%)	169 (19.3%)	0.381
**Patency period**	(days)	77.78 ± 54.69	72.44 ± 45.16	75.42 ± 50.58	0.534
**Previous COVID-19 exposure**	Yes	317 (72.2%)	300 (69%)	617 (70.6%)	0.293

^1^ Chi-squared test and Student’s *t*-test were used with a significance level of <0.05.

**Table 6:** Prevalence of the general side effects of Pfizer–BioNTech COVID-19 vaccine among healthcare workers in the Czech Republic, January–February 2021.

Variable	Outcome	≤43 Years Old	>43 Years Old	Total	Significance ^1^
**Side Effect**	**Injection site pain**	388 (93.3%)	343 (86.2%)	731 (89.8%)	**0.001**
	**Fatigue**	274 (65.9%)	232 (58.3%)	506 (62.2%)	**0.026**
	**Headache**	211 (50.7%)	160 (40.2%)	371 (45.6%)	**0.003**
	**Muscle pain**	170 (40.9%)	132 (33.2%)	302 (37.1%)	**0.023**
	Chills	153 (36.8%)	123 (30.9%)	276 (33.9%)	0.077
	Joint pain	119 (28.6%)	107 (26.9%)	226 (27.8%)	0.584
	Injection site swelling	108 (26.0%)	100 (25.1%)	208 (25.6%)	0.785
	Injection site redness	106 (25.5%)	81 (20.4%)	187 (23.0%)	0.082
	**Feeling unwell**	108 (26.0%)	79 (19.8%)	187 (23%)	**0.038**
	Fever	101 (24.3%)	76 (19.1%)	177 (21.7%)	0.073
	Lymphadenopathy	72 (17.3%)	60 (15.1%)	132 (16.2%)	0.388
	Nausea	61 (14.7%)	45 (11.3%)	106 (13.0%)	0.155
**Number of Side Effects**	**(1–12)**	4.50 ± 2.596	3.87 ± 2.599	4.19 ± 2.615	**0.001**
**Total**		416 (94.8%)	398 (91.5%)	814 (93.1%)	0.056
**Duration**	1 day	196 (47.2%)	168 (42.7%)	364 (45.1%)	
	3 days	159 (38.3%)	130 (33.2%)	289 (35.8%)	
	5 days	31 (7.5%)	45 (11.5%)	76 (9.4%)	
	1 week	17 (4.1%)	26 (6.6%)	43 (5.3%)	
	>1 week	8 (1.9%)	16 (4.1%)	24 (3.0%)	
	>1 month	4 (1.0%)	7 (1.8%)	11 (1.4%)	
**Severe Side Effects**		5 (1.1%)	6 (1.4%)	11 (1.3%)	0.747

^1^ Chi-squared test and ANOVA were used with a significance level of <0.05.

**Table 7:** Prevalence of oral side effects of Pfizer–BioNTech COVID-19 vaccine among healthcare workers in the Czech Republic, January–February 2021.

Variable	Outcome	≤43 Years Old	>43 Years Old	Total	Significance ^1^
**Side Effect**	Blisters	22 (37.3%)	19 (34.5%)	41 (36%)	0.760
	Halitosis	17 (28.8%)	12 (21.8%)	29 (25.4%)	0.391
	Ulcers	10 (16.9%)	6 (10.9%)	16 (14.0%)	0.354
	Bleeding gingiva	8 (13.6%)	5 (9.1%)	13 (11.4%)	0.453
	White/red plaque	6 (10.2%)	6 (10.9%)	12 (10.5%)	0.898
	Burning gingiva	5 (8.5%)	5 (9.1%)	10 (8.8%)	0.907
	Angular cheilitis	2 (3.4%)	3 (5.5%)	5 (4.4%)	0.591
	Tongue tingling	2 (3.4%)	3 (5.5%)	5 (4.4%)	0.591
	Taste disturbance	1 (1.7%)	3 (5.5%)	4 (3.5%)	0.276
	Vesicles	2 (3.4%)	2 (3.6%)	4 (3.5%)	0.943
	Swollen lips	3 (5.1%)	1 (1.8%)	4 (3.5%)	0.344
	Xerostomia	1 (1.7%)	2 (3.6%)	3 (2.6%)	0.518
**Number of Side Effects**	(1–12)	1.41 ± 0.098	1.22 ± 0.056	1.32 ± 0.603	0.093
**Total**		59 (13.4%)	55 (12.6%)	114 (13%)	0.727
**Onset**	1–3 days post-vaccination	10 (17.2%)	20 (37%)	30 (26.8%)	
	1st week post-vaccination	19 (32.8%)	13 (24.1%)	32 (28.6%)	
	2nd week post-vaccination	11 (19%)	7 (13.0%)	18 (16.1%)	
	3rd week post-vaccination	11 (19%)	10 (18.5%)	21 (18.8%)	
	4th week post-vaccination	7 (12.1%)	4 (7.4%)	11 (9.8%)	
**Location of ulcers, vesicles, and blisters**	Lips	20 (69.0%)	20 (80.0%)	40 (74.1%)	0.356
	Labial/buccal mucosa	6 (20.7%)	2 (8.0%)	8 (14.8%)	0.191
	Tongue	6 (20.7%)	1 (4.0%)	7 (13%)	0.069
	Palate	4 (13.8%)	1 (4.0%)	5 (9.3%)	0.216
	Gingiva	4 (13.8%)	1 (4.0%)	5 (9.3%)	0.216
**Number of ulcers, vesicles, and blisters’ locations**	(1–5)	1.38 ± 0.728	1.00 ± 0.00	1.20 ± 0.562	**0.012**
	One location affected	21 (72.4%)	25 (100.0%)	46 (85.2%)	
	Two locations affected	6 (20.7%)	0 (0.0%)	6 (11.1%)	
	Three locations affected	1 (3.4%)	0 (0.0%)	1 (1.9%)	
	Four locations affected	1(3.4%)	0 (0.0%)	1 (1.9%)	
**Location of white/red plaque**	Tongue dorsum	5 (83.3%)	4 (66.7%)	9 (75.0%)	0.505
	Soft palate	1 (16.7%)	1 (16.7%)	2 (16.7%)	1.000
	Labial/buccal mucosa	0 (0.0%)	1 (16.7%)	1 (8.3%)	0.296

^1^ Chi-squared test and ANOVA were used with a significance level of <0.05.

**Table 8:** Prevalence of skin-related side effects of the Pfizer–BioNTech COVID-19 vaccine among healthcare workers in the Czech Republic, January–February 2021.

Variable	Outcome	≤43 Years Old	>43 Years Old	Total	Significance ^1^
**Side Effect**	Rash	17 (63.0%)	11 (61.1%)	28 (62.2%)	0.900
	Urticaria	7 (25.9%)	3 (16.7%)	10 (22.2%)	0.464
	Other	4 (14.8%)	5 (27.8%)	9 (20.0%)	0.287
**Number of Side Effects**	(1–3)	1.03 ± 0.183	1.05 ± 0.224	1.04 ± 0.198	0.774
**Total**		27 (6.2%)	18 (4.1%)	45 (5.2%)	0.181
**Location of skin-related side effects**	Upper limb	18 (66.7%)	9 (50.0%)	27 (60.0%)	0.264
	Chest/trunk	8 (29.6%)	7 (38.9%)	15 (33.3%)	0.519
	Lower limb	7 (25.9%)	3 (16.7%)	10 (22.2%)	0.464
	Face	5 (18.5%)	4 (22.2%)	9 (20.0%)	0.761
	Back	5 (18.5%)	3 (16.7%)	8 (17.8%)	0.874
**Number of locations**	(1–5)	1.59 ± 0.797	1.44 ± 0.856	1.53 ± 0.815	0.556

^1^ Chi-squared test and ANOVA were used with a significance level of <0.05.

**Table 9:** Correlation between medical anamneses and side effects of Pfizer–BioNTech COVID-19 vaccine among healthcare workers in the Czech Republic, January–February 2021.

		Age	Chronic Illnesses Number	Medical Treatments Number	General SE Number	General SE Duration	Oral SE Number	Oral SE Onset
Age	*r*	1	0.180 **	0.016	−0.180 **	0.097 **	−0.164	−0.202 *
	Sig.		0.003	0.756	0.000	0.005	0.086	0.033
	*n*	874	271	384	814	807	111	112
Chronic Illnesses Number	*r*	0.180 **	1	0.232 **	0.072	0.088	0.045	0.018
	Sig.	0.003		0.000	0.258	0.167	0.790	0.914
	*n*	271	272	249	249	246	38	37
Medical Treatments Number	*r*	0.016	0.232 **	1	0.108 *	0.122 *	0.175	0.168
	Sig.	0.756	0.000		0.041	0.021	0.188	0.208
	*n*	384	249	386	359	354	58	58
General SE Number	*r*	−0.180 **	0.072	0.108 *	1	0.256 **	0.202 *	−0.054
	Sig.	0.000	0.258	0.041		0.000	0.038	0.574
	*n*	814	249	359	817	809	106	109
General SE Duration	*r*	0.097 **	0.088	0.122 *	0.256 **	1	0.279 **	0.025
	Sig.	0.005	0.167	0.021	0.000		0.004	0.794
	*n*	807	246	354	809	809	106	107
Oral SE Number	*r*	−0.164	0.045	0.175	0.202 *	0.279 **	1	0.143
	Sig.	0.086	0.790	0.188	0.038	0.004		0.138
	*n*	111	38	58	106	106	111	109
Oral SE Onset	*r*	−0.202 *	0.018	0.168	−0.054	0.025	0.143	1
	Sig.	0.033	0.914	0.208	0.574	0.794	0.138	
	*n*	112	37	58	109	107	109	113

* Correlation is significant at the 0.05 level (2-tailed); ** Correlation is significant at the 0.01 level (2-tailed); SE, side effects.

**Table 10:** Number of doses and the side effects of Pfizer–BioNTech COVID-19 vaccine among healthcare workers in the Czech Republic, January–February 2021.

	One Dose	Two Doses	Significance ^1^
Injection site pain	44 (88%)	690 (90%)	0.657
Injection site swelling	12 (24%)	197 (25.7%)	0.791
**Injection site redness**	4 (8%)	183 (23.9%)	**0.010**
Fatigue	30 (60%)	477 (62.2%)	0.757
Headache	21 (42%)	353 (46%)	0.580
Nausea	6 (12%)	101 (13.2%)	0.812
Feeling unwell	16 (32%)	173 (22.6%)	0.125
Muscle pain	18 (36%)	286 (37.3%)	0.855
Chills	17 (34%)	260 (33.9%)	0.988
Joint pain	17 (34%)	210 (27.4%)	0.311
Fever	11 (22%)	168 (21.9%)	0.987
Lymphadenopathy	8 (16%)	124 (16.2%)	0.975
Number of General SE	4.08 ± 2.52	4.20 ± 2.63	0.752
Ulcers	1 (14.3%)	15 (13.9%)	0.977
Vesicles	0 (0%)	4 (3.7%)	0.604
Blisters	2 (28.6%)	39 (36.1%)	0.687
White/red plaque	1 (14.3%)	11 (10.2%)	0.731
**Halitosis**	4 (57.1%)	25 (23.1%)	**0.045**
Bleeding gingiva	2 (28.6%)	11 (10.2%)	0.137
Burning gingiva	0 (0%)	10 (9.3%)	0.399
Swollen lips	0 (0%)	4 (3.7%)	0.604
Angular cheilitis	0 (0%)	5 (4.6%)	0.561
Xerostomia	0 (0%)	3 (2.8%)	0.655
Taste disturbance	0 (0%)	4 (3.7%)	0.604
Tongue tingling	0 (0%)	5 (4.6%)	0.561
Number of Oral SEs	1.43 ± 1.13	1.31 ± 0.56	0.610
Rash	2 (100%)	26 (60.5%)	0.260
Urticaria	0 (0%)	10 (23.3%)	0.439
Other skin-related SEs	0 (0%)	9 (20.9%)	0.469
Number of skin-related SEs	1 ± 0	1.04 ± 0.202	0.774

^1^ Chi-squared test and ANOVA were used with a significance level of <0.05.

## Data Availability

The data that support the findings of this study are available from the corresponding author upon reasonable request.

## Supplementary Materials

The following are available online at https://www.mdpi.com/article/10.3390/jcm10071428/s1, Table S1: OSECV Instrument (in Czech).
