# Supplementary material for: Prevalence of COVID-19 Vaccine Side Effects among Healthcare Workers in the Czech Republic
Source: J Clin Med. 2021 Apr 1;10(7):1428. doi: 10.3390/jcm10071428 (PMC8037149; doi:10.3390/jcm10071428)
Supplement: Supplementary file 1 [file jcm-10-01428-s001.pdf]

Orální vedlejší účinky vakcín COVID-19: Multicentrická průřezová studie

Úvod

Tento projekt je průřezovou studií, která si klade za cíl vyhodnotit vedlejší účinky vakcín proti COVID-19, zejména vedlejší účinky, které mohou mít vliv na ústní dutinu. Cílovou populací jsou zdravotničtí pracovníci, kteří dostávají vakcínu proti COVID-19 v rané fázi očkování. Dotazník obsahuje 20 otázek, jejichž zodpovězení bude trvat přibližně 7 minut. Výstupem projektu je zlepšit povědomí a znalosti laické i vědecké komunity o bezpečnosti vakcín proti COVID-19.

Účast

- **Podmínky účasti**
  Dobrovolně se může zúčastnit každý zdravotnický pracovník po 30 dnech od první dávky vakcíny proti COVID-19. Zúčastnění dobrovolníci musí být starší 18 let.
- **Výhody**
  Za vyplnění dotazníku respondentovi nenáleží finanční ani jiná odměna.. Účast v této multicentrické studii poskytne vědecké důkazy a informace pro tvůrce politik na místní, národní a mezinárodní úrovni o bezpečnosti a krátkodobých vedlejších účincích vakcín proti COVID-19.
- **Rizika**
  Nejsou očekávány žádné fyzické či psychické vedlejší účinky, ani nehrozí žádné riziko plynoucí z účasti v dotazníkovém průzkumu.

Ochrana dat

Správcem získaných dat je Masarykova univerzita (MUNI) a data budou použita univerzitou výhradně pro účely výzkumu v oblasti veřejného zdraví. Data nebudou zpřístupněna ani použita jinými institucemi ani nebudou využita pro jiné účely. Získaná data budou zpracována a analyzována po dobu trvání projektu (přibližně jeden rok). Po dokončení projektu budou získaná data zašifrována a bezpečně uložena prostřednictvím univerzity v rámci opatření k zajištění integrity výzkumu.

Kontakt

Máte-li dotazy týkající se studie, kontaktujte nás.

Dr. Abanoub RIAD, DDS

Adresa: Masarykova Univerzita, Lékařská fakulta
Pavilon A21/315 – Kamenice 753/5, 625 00 Brno, Česká republika

Email: abanoub.riad@med.muni.cz
Tel: +420549496572

Kliknutím na tlačítko “DOTAZNÍK” níže prohlašuji, že je mi alespoň 18 let a souhlasím s účastí ve výzkumné studii. Účastním se dobrovolně a souhlasím, aby byly data z této studie použity ve vědeckých publikacích. .

DOTAZNÍK

1. Demografické údaje	
**I.** Pohlaví	○ Žena<br>○ Muž<br>○ Nebinární<br>○ Nepřeji si uvést
**II.** Věk	○ Nabídka s čísly (18-99)
**III.** Povolání	○ Lékař (MUDr.)<br>○ Zubní lékař (MDDr., MUDr.)<br>○ Všeobecná sestra<br>○ Praktická sestra<br>○ Porodní asistentka<br>○ Fyzioterapeut<br>○ Jiné *(prosím upřesněte)*
V případě lékaře, doplňte specializaci	○ Alergologie<br>○ Anesteziologie<br>○ Kardiochirurgie<br>○ Kardiologie<br>○ Kardiotorakální chirurgie<br>○ Chemická patologie<br>○ Dětská a dorostová psychiatrie<br>○ Klinická onkologie<br>○ Klinická farmakologie<br>○ Klinická radiologie<br>○ Dermatovenerologie<br>○ Urgentní medicína<br>○ Endokrinologie<br>○ Gastroenterologie<br>○ Interní medicína<br>○ Všeobecná psychiatrie<br>○ Všeobecná chirurgie<br>○ Geriatrie<br>○ Hematologie<br>○ Histopatologie<br>○ Imunologie<br>○ Infekční nemoci<br>○ Maxilofaciální chirurgie<br>○ Lékařská mikrobiologie a virologie<br>○ Nefrologie<br>○ Neurologie<br>○ Neuropsychiatrie<br>○ Neurochirurgie<br>○ Nukleární medicína (radiační onkologie)<br>○ Gynekologie a porodnictví<br>○ Pracovní lékařství<br>○ Oftalmologie<br>○ Ortopedie<br>○ Otorinolaryngologie<br>○ Dětská chirurgie<br>○ Pediatrie<br>○ Rehabilitační lékařství

	o Plastická chirurgie<br>o Veřejné zdravotnictví<br>o Pneumologie<br>o Revmatologie<br>o Urologie<br>o Cévní chirurgie<br>o Jiná *(prosím upřesněte)*
**IV.** Země kde proběhla vakcinace	o Česká republika<br>o Německo<br>o Slovenská republika<br>o Turecko
**V.** Délka profesní praxe	o 0-99
**2. Zdravotní anamnéza**	
**VI.** Léčíte se s chronickým onemocněním?	o Ano<br>o Ne
Pokud jste odpověděli ano, zvolte alespoň jednu z následujících možností:	o Astma<br>o Onemocnění krve<br>o Onemocnění kostí<br>o Onemocnění střev<br>o Onkologické onemocnění<br>o Onemocnění srdce<br>o Chronická hypertenze<br>o Chronická obstrukční plicní nemoc (CHOPN)<br>o Diabetes mellitus typ I<br>o Diabetes mellitus typ II<br>o Onemocnění ledvin<br>o Revmatoidní artritida<br>o Jiné *(prosím upřesněte)*
**VII.** Užíváte v současnosti nějaké léky?	o Ano<br>o Ne
**VIII.** Pokud jste odpověděli ano, zvolte alespoň jednu z následujících možností:	o Antibiotika<br>o Antidepresiva<br>o Antihistaminika<br>o Imunosupresivní léky<br>o Běžná analgetika<br>o Nesteroidní antirevmatika (NSAID)<br>o Opiátová analgetika<br>o Antiepileptika<br>o Jiné *(prosím upřesněte e)*
**3. Anamnéza související s očkováním proti COVID-19**	
**IX.** Datum první dávky očkování:	o Zvolte datum z (kalendáře)
**X.**<br>Aplikovali Vám již i druhou dávku?	o Ano<br>o Ne
**XI.** Typ očkovací látky:	o vakcína Pfizer-BioNTech COVID-19<br>o vakcína Moderna COVID-19<br>o vakcína Oxford–AstraZeneca COVID-19<br>o vakcína Sinovac<br>o vakcína Sputnik
**XII.** Byl Vám diagnostikován COVID-19?	o Ano<br>o Ne

Pokud jste odpověděli ano, zvolte datum uzdravení.	o Zvolte datum z (kalendáře)
**XIII.** Byli jste v kontaktu s osobou s onemocněním COVID-19 potvrzeným PCR-testem?	o Ano<br>o Ne
**4. Vedlejší účinky očkování**	
**XIV.** Vyskytly se u vás některé z následujících orálních (ústních) příznaků během čtyř týdnů po očkování?	o Vředy<br>o Vezikuly)<br>o Opary<br>o Bílý nebo červený plak<br>o Nepříjemný zápach z úst<br>o Krvácení dásní<br>o Pálení dásní<br>o Otok rtů<br>o Jiné *(*prosím upřesněte*)*
Pokud jste označili vředy, vezikuly nebo opary, zvolte alespoň jednu z následujících oblastí:	o Jazyk<br>o Patro<br>o Labiální nebo bukální sliznice<br>o Dásně<br>o Rty
Pokud jste označili bílý nebo červený plak, zvolte alespoň jednu z následujících oblastí:	o Dorsum jazyka<br>o Měkké patro<br>o Labiální nebo bukální sliznice
**XV.** Kdy se tyto orální příznaky objevily?	o 1-3 dny po očkování<br>o Během prvního týdne po očkování<br>o Během druhého týdne po očkování<br>o Během třetího týdne po očkování<br>o Během čtvrtého týdne po očkování
**XVI.** Vyskytly se u Vás během čtyř týdnů po očkování některé z uvedených příznaků?	o Bolest v místě vpichu<br>o Otok v místě vpichu<br>o Zarudnutí v místě vpichu<br>o Únava<br>o Bolest hlavy<br>o Nevolnost<br>o Pocit nepohody<br>o Bolest svalů<br>o Zimnice<br>o Bolest kloubů<br>o Horečka<br>o Zduřené mízní uzliny (lymfadenopatie)<br>o Žádné
**XVII.** Pokud jste označili některý z předešlých příznaků, zvolte délku jeho trvání:	o 1 den<br>o 3 dny<br>o 5 dnů<br>o 1 týden<br>o > 1 týden<br>o > 1 měsíc
**XVIII.** Vyskytly se u vás během čtyř týdnů po očkování nějaký závažný příznak vyžadující hospitalizaci, např. dýchací	o Ano<br>o Ne

obtíže, otok obličeje a krku, zrychlený pulz, horečka nebo generalizovaná vyrážka (celotělový výsev)?	
**XIX.** Vyskytly se u Vás během čtyř týdnů po očkování některé z uvedených kožních příznaků?	o Vyrážka<br>o Kopřivka<br>o Angioedém<br>o Jiné *(*prosím upřesněte *e)*
Pokud jste odpověděli vyrážka, kopřivka nebo angioedém, zvolte postiženou oblast:	o Obličej<br>o Horní končetina<br>o Dolní končetina<br>o Hrudník / trup<br>o Záda

Odeslat
